# Broad Beam Plasma Enhanced Low-Temperature Growth of Oriented Aluminum Nitride Thin Films

**DOI:** 10.1021/acsami.5c13449

**Published:** 2025-11-04

**Authors:** Yifan Liu, Keliang Wang, Tyler Johnson, Aniwat Juhong, Junwoo Lee, Bo Li, Shi-You Ding, Zhen Qiu, Qi Hua Fan

**Affiliations:** 1 Department of Electrical and Computer Engineering, 3078 Michigan State University, East Lansing, Michigan 48824, United States; 2 Institute for Quantitative Health Science and Engineering, Michigan State University, East Lansing, Michigan 48824, United States; 3 Fraunhofer USA Center Midwest, East Lansing, Michigan 48824, United States; 4 Department of Chemical Engineering and Materials Science, 3078 Michigan State University, East Lansing, Michigan 48824, United States; 5 Department of Plant Biology, 3078 Michigan State University, East Lansing, Michigan 48824, United States; 6 Department of Biomedical Engineering, 3078 Michigan State University, East Lansing, Michigan 48824, United States

**Keywords:** aluminum nitride, thin film, ion source, low-temperature growth, piezoelectric response

## Abstract

Aluminum nitride (AlN) thin films with (0002) orientation have exceptional piezoelectric and optoelectronic properties for various applications. It remains challenging to grow highly oriented AlN films at low temperature (e.g., below 200 °C) using conventional magnetron sputtering. This study introduces a broad beam ion source-enhanced pulsed DC magnetron sputtering, which enables the growth of (0002) preferentially oriented polycrystalline AlN films at room temperature. The effects of the ion energy and ion flux on surface roughness, crystal orientation, and piezoelectric properties are systematically studied. The film crystallinity is significantly improved under an optimum ion energy; X-ray diffraction shows that the full width at half-maximum (FWHM) of the (0002) peak decreases from 0.7298 to 0.3751°. The average surface roughness is reduced from 2.65 to 0.95 nm. The effective piezoelectric *d*_33_^*eff*^ value increases from 1.69 to 6.06 pm/V. These findings demonstrate that the ion beam facilitates crystal growth under nonthermal equilibrium conditions, offering significant advantages over conventional thin film growth.

## Graphical abstract

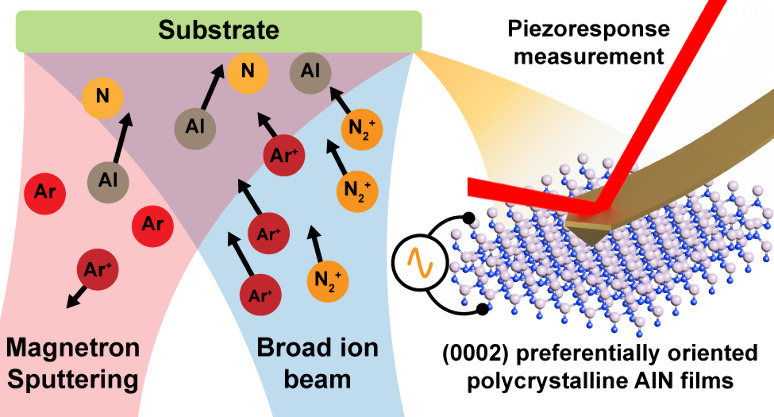

## 1 Introduction

Aluminum nitride (AlN) is a group III–V compound semiconductor with a stable wurtzite structure. AlN thin films exhibit a range of unique properties, such as a large bandgap (∼6.2 eV), high electrical resistivity (10^11^–10^14^ Ω·cm) and high thermal conductivity (320 W/m·K), good effective piezoelectric coefficient (5.3 pm/V), high acoustic wave velocity (>5500 m/s), and excellent chemical stability.^1,2^ These properties make AlN an attractive material for various applications, such as deep ultraviolet (UV) optoelectronics,^3^ piezoelectric material for microelectromechanical systems (MEMS)^4^ and surface acoustic wave devices,^5^ resonating components in RF-MEMS oscillators,^6^ and hard coatings.^7^ Many of these properties are closely related to the (0002) orientation along the c-axis perpendicular to the film surface.^8–10^

AlN thin films can be fabricated using various methods, including chemical vapor deposition,^11,12^ molecular beam epitaxy,^13^ pulsed laser deposition,^14^ and magnetron sputtering.^15,16^ Although each method has unique advantages, magnetron sputtering is attractive for its simplicity, scalability, and applicability in both academic research and industrial production. However, most reported AlN thin films require elevated temperatures (∼300 °C) or additional underlying metal layer to achieve (0002) oriented crystallization.^11–19^ Recently, efforts have been made to deposit AlN on flexible substrates to fabricate MEMS device using magnetron sputtering.^20,21^ However, high deposition temperature limits the use of heat-sensitive substrates such as flexible polyethylene terephthalate (PET) sheets, poses challenges to the operation of large-scale vacuum deposition systems and increases manufacturing costs.

A lot of efforts have been devoted to reducing AlN thin film growth temperature with magnetron sputtering. Kar et al. reported a growth temperature of ∼ 200 °C.^22^ In 2015, two groups achieved (0002) oriented polycrystalline AlN deposition without external heating using high power impulse magnetron sputtering.^23,24^ In 2016, Yarar et al. demonstrated (0002) oriented AlN deposition on nominally unheated substrates using direct-current (DC) magnetron sputtering.^25^ Then in 2022, Bakri et al. also achieved (0002) oriented AlN by radio frequency (RF) magnetron sputtering at room temperature.^26^ Perez et al. demonstrated low-temperature deposition of (0002) oriented, high-thermal-conductivity AlN using DC magnetron sputtering.^27^ However, these previous works have notable limitations, such as requiring seed layers (e.g., Pt or Mo), low deposition rates, relatively low piezoelectric coefficients with a measured *d*_33_^*eff*^ value of <5.6 pm/V or lacking scalability for mass production. As an alternative method, this work presents a broad beam ion source enhanced pulsed DC magnetron sputtering (BIS-DCMS) method, enabling direct growth of (0002) oriented AlN polycrystalline thin films on glass and silicon substrates at room temperature.

Ion sources are plasma generation devices that emit ion beams to interact with the atoms of deposited surface, thereby modulating the thin film microstructure.^28–33^ Two types of commonly used ion sources for surface treatments are filament-based and anode-layer ion sources. While the filament-type ion sources can emit ion beams with widely tunable ion energy, they are incompatible with reactive gases, such as oxygen. The anode-layer ion sources, on the other hand, require a high discharge voltage of >250 V to sustain the plasma, resulting in high ion energy that may damage the film/substrate interface. Additionally, the ion flux density is proportional to the ion energy in anode-layer ion sources, which limits the independent control of these parameters. Such control is essential for optimizing ion-surface interactions to promote thin-film crystallization without damaging the interface.

The broad beam ion source used in this study has unique characteristics, which overcomes the limitations of conventional ion sources. It is particularly suitable for low-temperature deposition of dense, high-quality thin films.^34–36^ It enables independent control of the ion energy and ion flux density in the ion energy range of 10 to 200 eV, allowing optimum ion energy transfer to the film for preferential crystal orientation growth. Furthermore, the broad beam ion source is compatible with reactive gases. It can be easily integrated into existing magnetron sputtering systems, providing a scalable solution for depositing high-quality thin films.

## 2 Results

### 2.1 Broad Beam Ion Source Enhanced Pulsed DC Magnetron Sputtering (BIS-DCMS)

Figure 1 illustrates the setup for the broad beam ion source enhanced pulsed DC magnetron sputtering system. Inside the sputtering chamber, a substrate holder is mounted on the top, while the magnetron and ion source are positioned on the bottom, both oriented at a 45-degree angle toward the substrate holder as shown in Figure 1(a). The operation principle of the ion source is illustrated in Figure 1(b). The ion source consists of a broad anode with a magnetic field distributed above its surface. The surrounding cathode intercepts a substantial portion of the magnetic flux, enabling effective confinement of the energetic electrons to sustain the plasma discharge. A positive bias voltage is applied to the anode relative to the cathode to ignite plasma. As electrons accelerate toward the anode, they experience a Lorenz force and subsequently drifts along the E × B direction above the anode surface. Under steady-state discharge conditions, a gradual drop of the electric potential from the anode to the substrate, establishing an electric field that drives positively charged ions as a single broad beam toward the substrate. The ion source discharge can be sustained by DC power and/or RF power. The strong confinement of the electrons by the magnetic field across the anode surface results in a high ion flux density.

**Figure 1:**
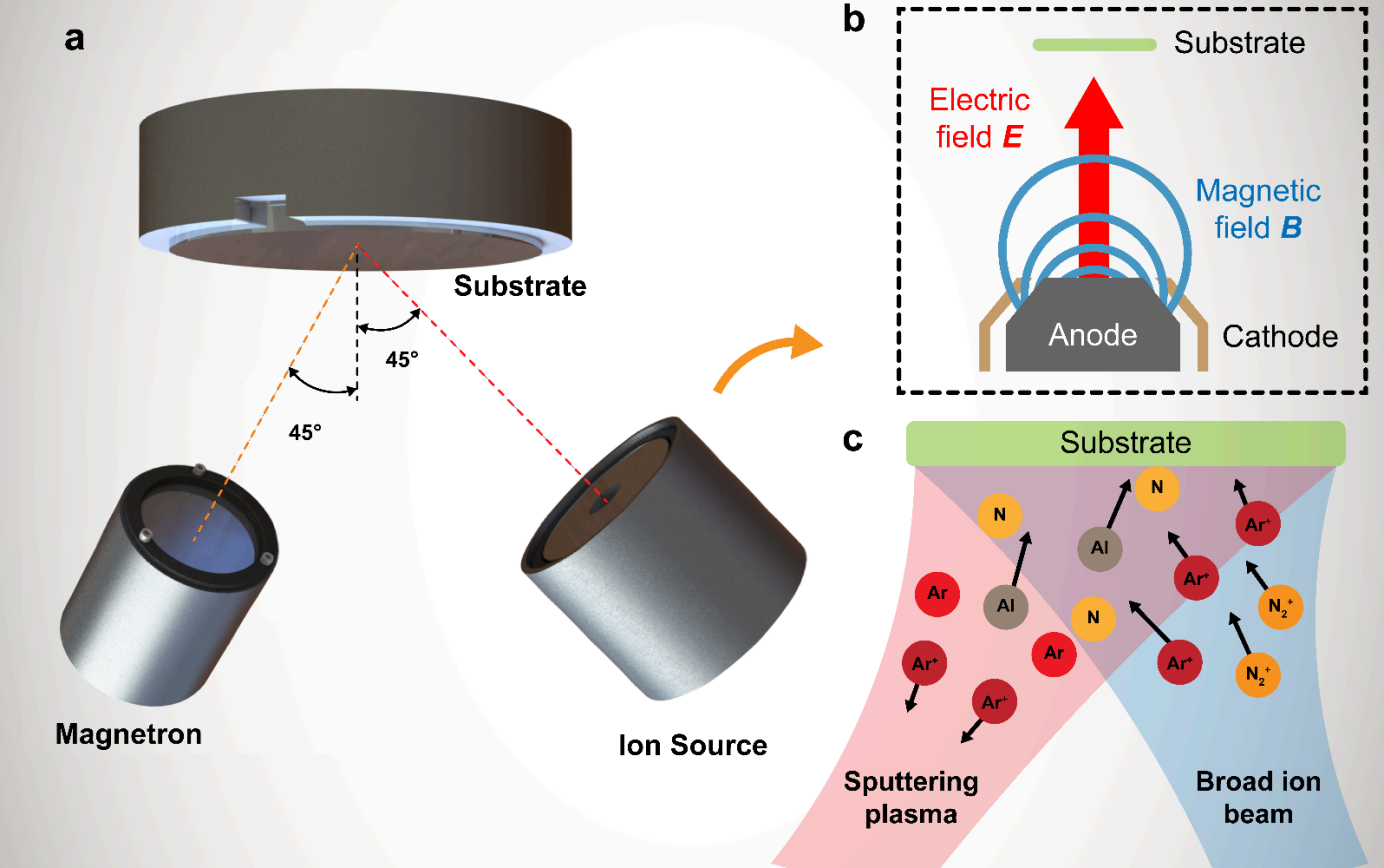
Deposition system overview. (a) Conceptual illustration of the broad beam ion source enhanced pulsed DC magnetron sputtering system with both the magnetron and ion source facing the substrate at a 45° angle. (b) Side view of the broad beam ion source, illustrating its operation principle. (c) Illustration of reactive DC magnetron sputtering deposition of AlN film under the assistance of the broad beam ion source.

The AlN film deposition under the assistance of the broad beam ion source is illustrated in Figure 1(c). Al atoms sputtered off the metal target react with the reactive nitrogen species in the gas phase or on the substrate surface to form AlN. Then the ion source generates a beam of ions with controlled energy (i.e., Ar^+^ and N_2_^+^) toward the substrate, providing additional energy to enhance the adatom mobility and promote AlN film crystallization.

As mentioned above, the broad beam ion source used in this work overcomes the problem of conventional ion source by utilizing a combined RF and DC voltage to independently control the ion energy and ion flux density. This unique characteristic is presented in Figure 2. Figure 2(a) illustrates discharge images of the ion source operating alone and simultaneously with a sputtering magnetron. The ion beam is approximately 80 mm in diameter.

**Figure 2:**
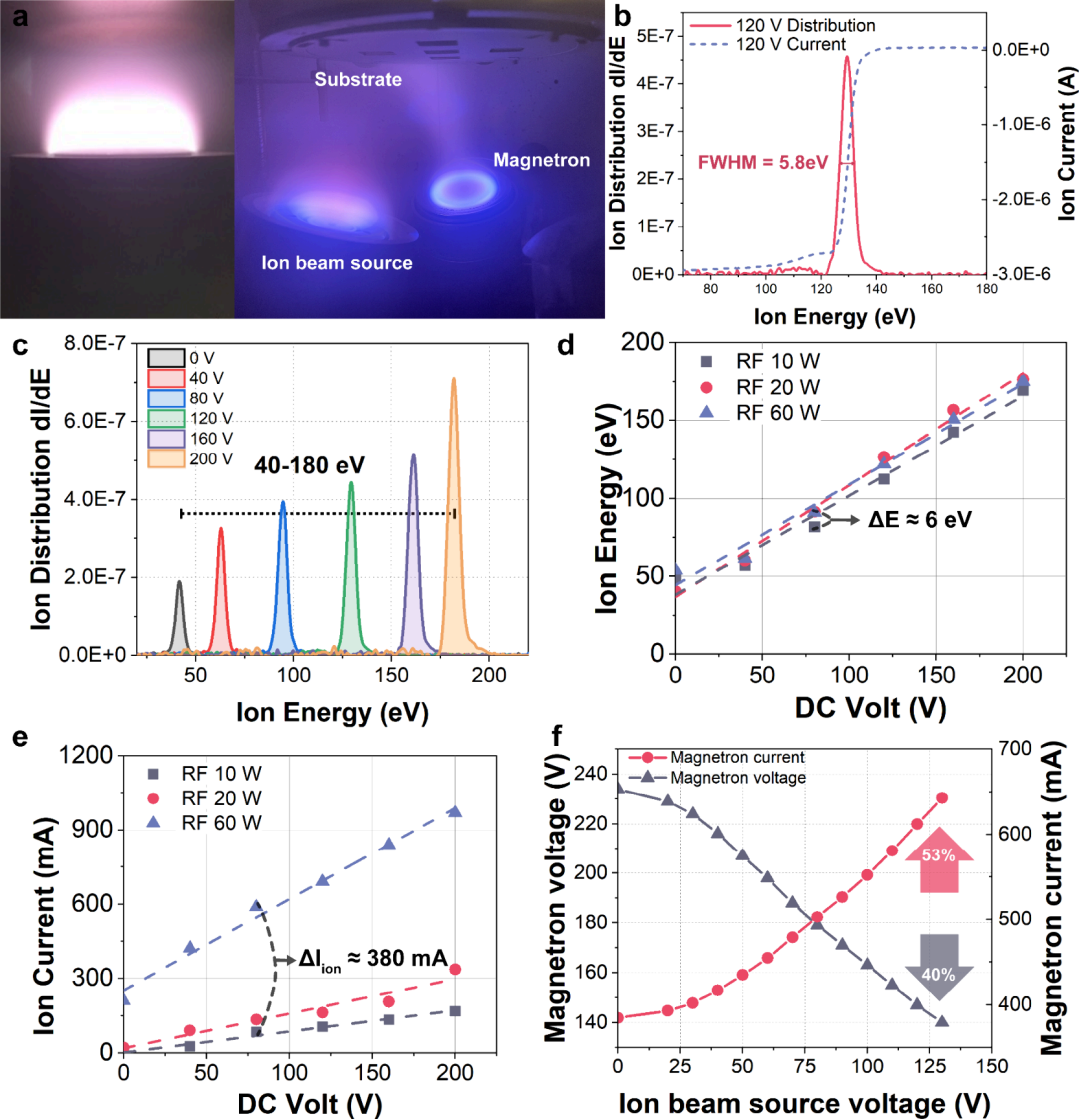
Discharge characteristics of the broad beam ion source. (a) Left: stable discharge image of the ion source excited by RF + DC voltage. Right: Discharge image of a sputtering magnetron and the ion source operating simultaneously. (b) Ion beam current and ion energy distribution under the excitation of 120 V DC voltage and 60 W RF power. (c) Ion energy distribution under different DC voltages with an RF power of 60 W. (d) Ion energy and (e) ion current as a function of applied DC voltage and RF powers. (f) Magnetron discharge voltage and current modulated by the ion source voltage.

Figure 2(b) illustrates the ion current and ion energy distribution when it is excited by a DC voltage of 120 V and RF power of 60 W. The ion energy exhibits a narrow peak distribution, with a FWHM of only 5.8 eV and a sharp cutoff, enabling precise control of the energy transferred to the film. Figure 2(c) demonstrates that the ion energy can be controlled at any desired level between 40 to 180 eV by varying DC voltage from 0 to 200 V with an RF power of 60 W. The ion energy can be further reduced by adjusting the RF power. This precisely controlled ion energy results in optimum ion-film interactions; low ion energies are insufficient in enhancing film crystallization and high ion energies may damage the film.

Using combined RF and DC powers to excite the ion source enables the decoupling of ion current and ion energy. As shown in Figure 2(d), the RF power has minimal influence on ion energy when the DC voltage is held constant. When the DC voltage was fixed at 80 V, the ion energy varies by only 6 eV as the RF power increases from 10 to 60 W. In contrast, ion energy exhibits an approximately linear relationship with the DC voltage. Conversely, under a fixed DC voltage, the ion current can be modulated by the RF power as shown in Figure 2(e). Specifically, when the DC voltage is maintained at 80 V, increasing the RF power from 10 to 60 W results in an ion current increase of approximately 380 mA. These characteristics allow independent evaluation of the effects of ion energy and ion flux density, which is crucial to accurately controlling the film growth and microstructure. In contrast, the ion energy and ion flux density are typically coupled in conventional ion sources; they change proportionally.

Furthermore, the ion source and magnetron discharges mutually enhance each other. Electrons from the negatively biased magnetron promote the ion source discharge and the ions from the positively biased ion source increase the magnetron plasma density. This interaction results in a significantly increased magnetron discharge current, as shown in Figure 2(f). With the sputtering power is kept constant, increasing the ion source voltage from 0 to 130 V causes the magnetron voltage to decrease accordingly by ∼ 40%.

The coupled ion source and magnetron discharge results in a unique “soft sputtering mode” with two distinct advantages over conventional DC magnetron sputtering. First, the film deposition rates are significantly increased due to the sublinear dependence of sputtering yield on the sputtering ion energy. Our research has demonstrated a 30% increase in the deposition rate under the same sputtering power. Second, the energetic sputtered atoms are largely reduced or even eliminated. In conventional DC magnetron sputtering, the discharge voltage often exceeds 250 V, resulting in sputtered atoms with an energy tail reaching approximately 200 eV according to the Thompson distribution.^37,38^ The energetic atoms could damage the film interface and create defects. A lower magnetron discharge voltage is therefore highly desirable but unachievable in conventional DC magnetron sputtering.

### 2.2 Ion Beam Enhanced Growth of AlN Thin Films

Using the BIS-DCMS, we deposited AlN thin films under various conditions, including with and without the ion source, and with different RF/DC input combinations for the ion source. X-ray diffraction was used to evaluate the crystallinity of AlN thin films prepared under different conditions. The results are presented in Figure 3, with detailed parameters summarized in Table S1.

**Figure 3:**
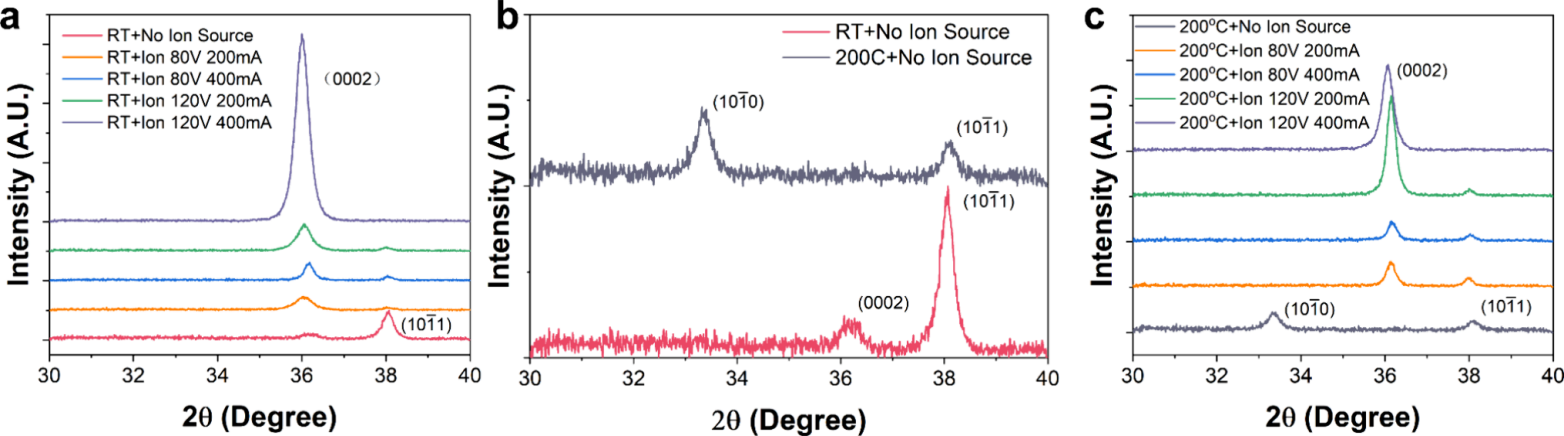
X-ray diffractogram of AlN films deposited by BIS-DCMS. (a) XRD diffractograms of AlN films deposited at room temperature with different parameters. (b) XRD diffractograms of AlN films without Ion source with or without external heating source. (c) XRD diffractograms of AlN films deposited at 200 °C with different parameters.

As shown in Figure 3(a), the AlN film deposited without the ion source only exhibited a small (0002) peak and a (101̅1) peak. However, when the ion beam was applied during deposition, the (101̅1) peak was largely reduced while the (0002) peak became more pronounced. Notably, under 120 V and 400 mA ion beam treatment, a large single (0002) peak was observed, indicating a preferential orientation of AlN crystals. It demonstrates that the 2θ scan FWHM of (0002) peak was reduced from 0.7298° to 0.3751° compared to the samples deposited without ion source.

The ion beam treatment resulted in a small increase in the substrate temperature, about 30 °C. To exclude the potential influence of the heating caused by the ion beam treatment during deposition, we deposited AlN films at a higher temperature of 200 °C using an external heater. The corresponding XRD results are presented in Figure 3(b), where the AlN films were deposited without the ion source at room temperature and 200 °C. Compared to the sample deposited at room temperature, the (101̅1) peak became lower while a small (101̅0) peak emerged when the film was deposited at 200 °C. In either case, no (0002) peak was observed. Hence, even 200 °C is insufficient to enhance the c-axis crystallization of AlN thin films using DC magnetron sputtering alone. In other words, the ion beam enhanced (0002) preferential orientation and crystallization of AlN thin films are realized under a nonthermal equilibrium condition.

Additional AlN thin films were deposited at 200 °C with ion beam assistance to further investigate the influence of substrate temperature. The XRD results are shown in Figure 3(c). The crystallization behavior is similar to the samples deposited at room temperature. The (0002) preferential orientation only occurred when the ion beam was at 120 V and 400 mA. Under this high ion energy and ion flux, the AlN film deposited at 200 °C has a slightly smaller 2θ scan FWHM of 0.3544° compared to 0.3751° for the film deposited with the same ion energy and ion flux at room temperature. It indicates that the temperature has minor influence on the deposited AlN films with ion beam treatment.

Based on the XRD result, we further analyzed the surface roughness of AlN films deposited at room temperature and 200 °C with and without ion source using atomic force microscopy (AFM). The results are displayed in Figure 4. Table S2 also lists details about the roughness measurement results. Among the films, sample 5 deposited with high ion energy (∼120 eV) and ion flux of 400 mA at room temperature exhibits the smoothest surface with an average roughness of 0.95 nm and a root-mean-square (RMS) roughness of 1.25 nm.

**Figure 4:**
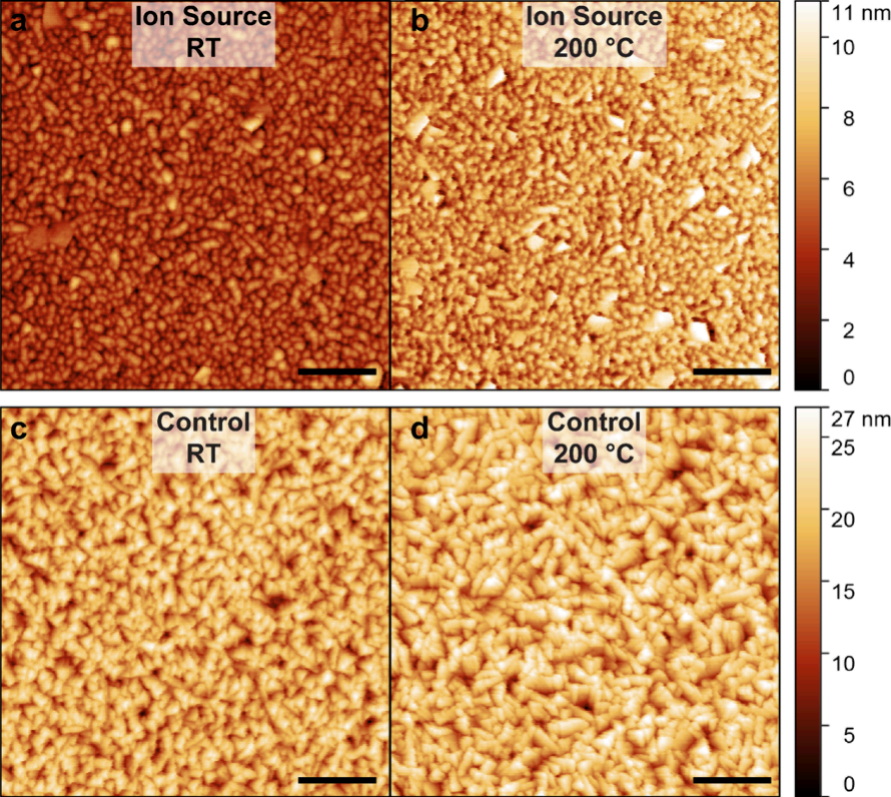
AFM images of AlN films deposited at (a) 120 V DC 400 mA ion current at room temperature. (b) 120 V DC 400 mA ion current at 200 °C. (c) Room temperature without ion source. (d) 200 °C without ion source. Scale bar: 200 nm.

As summarized in Table S2, AlN film deposited without the ion source at 200 °C shows a relatively rougher surface compared to the sample deposited at room temperature. This observation implies that the additional heating does not necessarily enhance the AlN thin film quality. In contrast, the ion beam-enhanced deposition resulted in significantly reduced roughness (e.g., samples 3, 5, 8, and 10).

The above studies were conducted using AlN films of 400 nm thickness. After clarifying the effects of the ion beam treatment on the AlN film surface roughness and crystallization, we further evaluated roughness, crystallization and piezoelectric coefficients of thicker AlN films of 1 μm for meaningful comparison with reported results. Figure 5 presents the AFM results of 1 μm thick AlN films deposited with and without the ion source. The average surface roughness values are 2.43 and 1.58 nm for the films deposited without and with the ion beam treatment.

**Figure 5:**
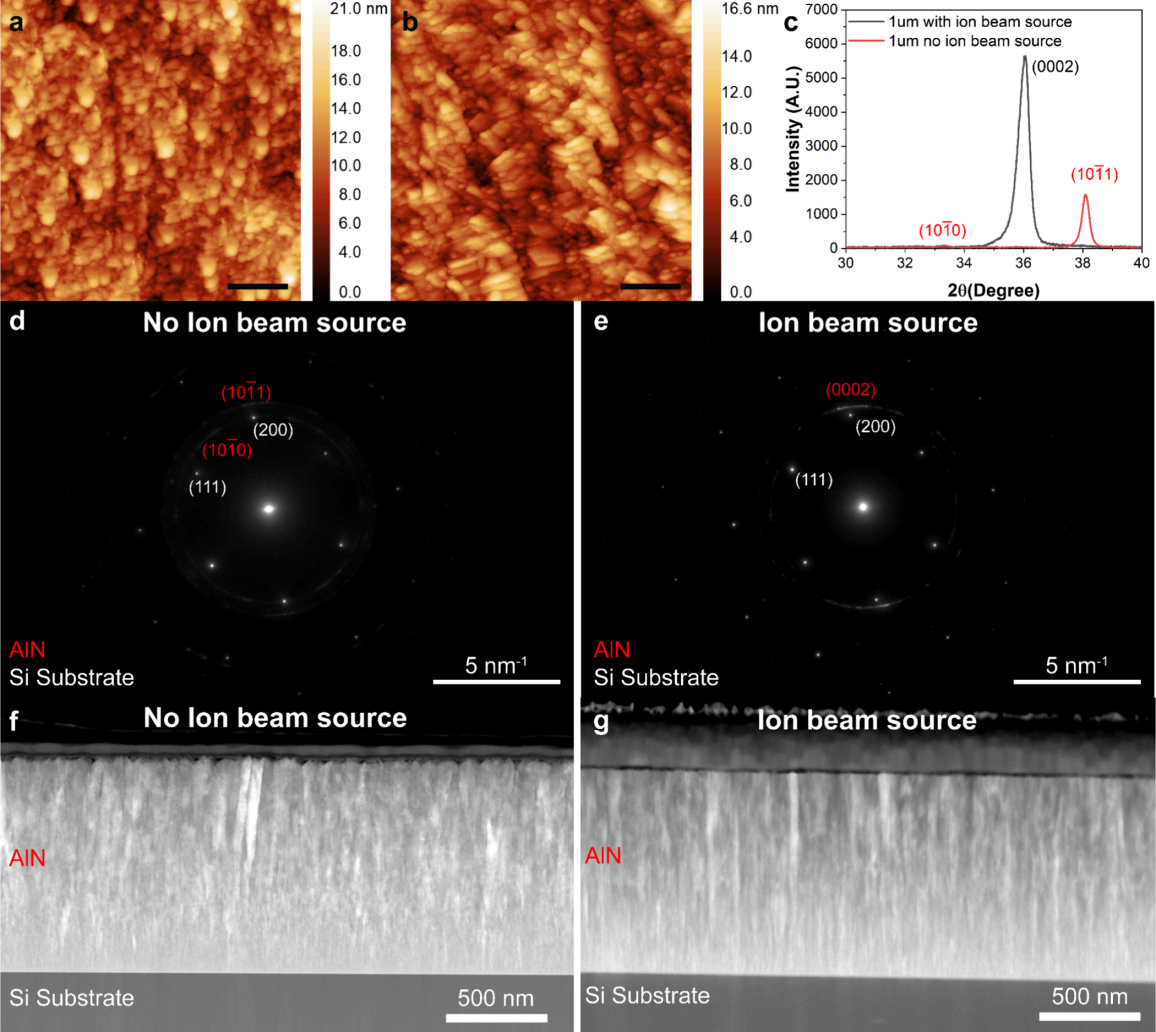
AFM images of AlN films of 1 μm thickness deposited at room temperature (a) without and (b) with the ion source. Scale bar: 200 nm. (c) XRD patterns of the AlN films shown in (a) and (b). Cross-sectional TEM diffraction patterns of the entire 1 μm-thick AlN film on silicon substrate, deposited (d) without and (e) with ion beam assistance. Cross-section TEM image of the entire 1 μm-thick AlN film on silicon substrate, deposited (f) without and (g) with ion beam assistance.

XRD measurements (Figure 5(c)) confirmed that the AlN film deposited with the ion source exhibited a shape (0002) peak, while the film deposited without the ion source showed small (101̅1) and (101̅0) peaks due to poor crystallization.

Additional diffraction results obtained using transmission electron microscopy (Figure 5(d) and (e)) further support our findings. The Transmission electron microscope (TEM) diffraction patterns were captured across the entire cross section of 1 μm-thick AlN thin films. To further analyze the crystallization behavior, we overlaid the diffraction rings from the silicon substrate and the AlN film. Without ion beam assistance, the AlN rings appear weaker and more diffuse. While the AlN film deposited with ion beam assistance exhibits sharper and more intense (0002) diffraction rings.

Furthermore, two additional TEM images covering the entire cross section of 1 μm-thick AlN thin films are presented in Figure 5(f) and (g). These images show that the surface of the AlN film deposited without ion beam assistance is obviously rougher than that of the ion beam-assisted sample. Additionally, the ion beam-assisted film exhibits more regular columnar grains oriented along the c-axis, suggesting uniform polycrystalline growth. These morphological observations further corroborate the results obtained from AFM, XRD, TEM diffraction and SEM image in Figure S1, indicating enhanced crystalline quality and a preferential orientation along the c-axis with the ion source.

The piezoelectric coefficient of thick AlN films was measured using a Piezoresponse Force Microscope experiment setup.^39^ A new set of samples using Si–Al–AlN layer structure was deposited, as shown in Figure 6(a). And the measured relationships between the piezoresponse amplitude and applied AC bias voltage are plotted as Figure 6(b). The background signal induced by the Al layer was also recorded to determine the actual piezoresponse property related to the AlN film. After removing the background signal, the measured d_33_ are 10.46 and 2.92 pm/V for the deposited AlN thin film with and without ion source. Then we can evaluate the effective piezoelectric coefficient *d*_33_^*eff*^ by calculating^40^ as eq 1:

$$d_{33}^{eff}=d_{33}-\frac{2S_{13}}{S_{11}+S_{12}}d_{31}$$ 1

Where the S_11_, S_12_, and S_13_ for AlN could be taken from reference^41^ as 3 × 10^–12^ m^2^/N, 8 × 10^–12^ m^2^/N, and 8 × 10^–12^ m^2^/N. And transverse coefficient d_31_ could be approximated as half of the effective piezoelectric coefficient following the convention in prior work.^42^ Based on this analysis, the effective piezoelectric coefficients for 1 μm AlN film deposited with and without ion source were calculated to be 6.06 pm/V and 1.69 pm/V. These results validate the capability of the BIS-PDMS to enhance thin film densification and crystallization, thereby improving piezoelectric performance.

**Figure 6:**
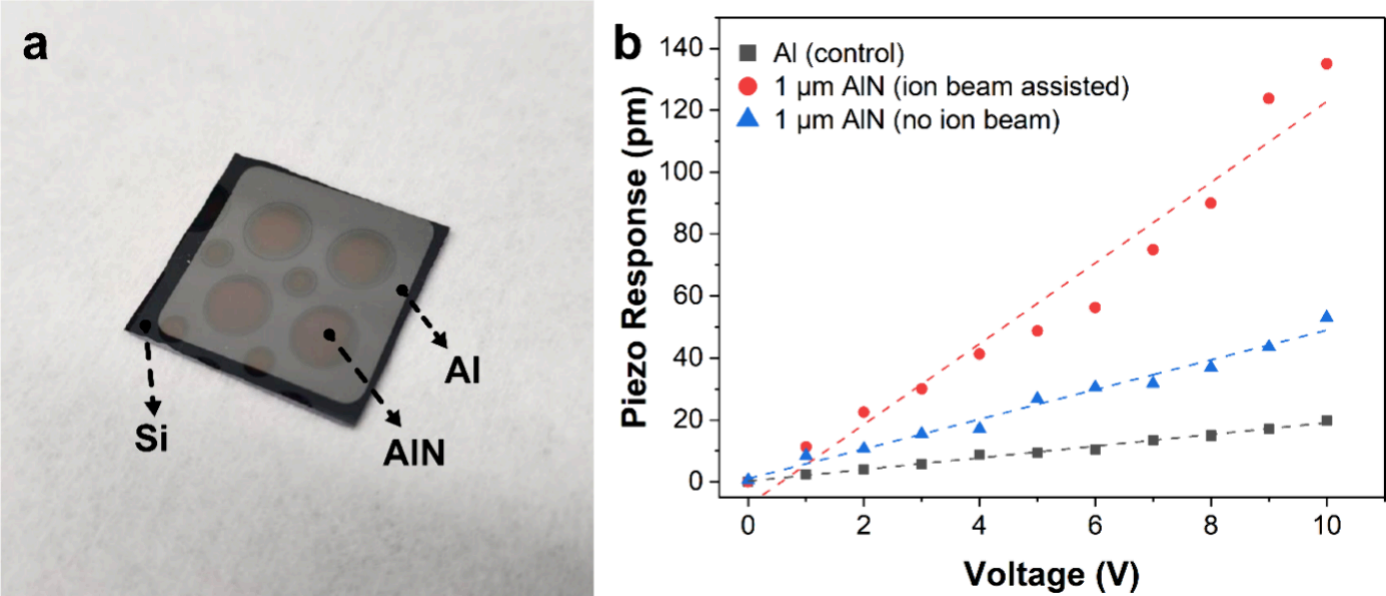
Piezoelectric property measurement of 1 μm-thick AlN films. (a) Image of the deposited Si–Al–AlN sample. (b) Measured relationship between piezoresponse amplitude and the applied AC bias voltage amplitude.

## 3 Conclusions

A broad beam ion source is used to enhance magnetron sputtering deposition of AlN thin films at room temperature. The ion energy and ion flux can be independently tuned, allowing optimum ion-film interactions to modulate the AlN thin film microstructure and properties. The ion beam treatment enables the growth of AlN films with preferential (0002) orientation at room temperature. Compared to the films deposited without the ion source, the FWHM of X-ray diffraction peak was reduced from 0.7298° to 0.3751° and the surface roughness is reduced from 2.65 to 0.95 nm. Most importantly, the effective piezoelectric coefficient *d*_33_^*eff*^ significantly increases from 1.69 to 6.06 pm/V with the ion beam-enhanced deposition. These findings demonstrate that the broad beam ion source offers unique advantages when combined with conventional magnetron sputtering and has many potential applications for thin film growth.

## 4 Methods

A broad beam plasma source (model SPR-100, Scion Plasma LLC) was used to enhance the growth of AlN thin films. A circular magnetron (model TORUS TM3, K. J. Lesker) was used for the sputtering deposition of AlN films, utilizing a pure aluminum target (99.99%) with a diameter of 76.2 mm and a thickness of 6.35 mm. The substrates used were glass and silicon of 25.4 by 25.4 mm square in each run. The distance from both the circular magnetron and the broad beam plasma source to the substrates was set to 100 mm. Prior to deposition, the substrates were ultrasonically cleaned in acetone for 10 min, rinsed sequentially in isopropyl alcohol and deionized water, and dried in an oven at 80 °C for at least 1 h. They were then mounted onto a substrate holder and transferred into a vacuum chamber for thin film deposition.

AlN thin film deposition was conducted using a PVD75 system (K. J. Lesker), which has a loadlock for sample transfer without breaking the vacuum. The system base pressure was maintained below 6 × 10^–5^ Pa before each deposition. The process gases included 9 sccm Ar and 3 sccm N_2_, establishing a chamber pressure of 0.25 Pa by a throttle valve. The magnetron sputtering was conducted using a pulse DC power supply, with a constant sputtering power of 80 W, a pulse frequency of 100 kHz, and a reverse time of 1 μs. Additionally, a substrate heating platform with PID control was employed to maintain the substrate at a specific temperature.

The ion source was excited with a 13.56 MHz RF power supply in a range of 5–200 W combined with a DC power supply with the voltage adjustable in the range of 0–250 V. The ion energy and ion flux density of the ion source were measured using a plasma ion analyzer (Semion 2500, Impedans) with the detector set on the substrate holder.

Deposition rates were initially evaluated by depositing thick films over extended durations, and the deposition times were subsequently adjusted to produce AlN thin films of approximately 400 nm or 1 μm thickness for consistent comparisons. All the film depositions were conducted at room temperature except otherwise specified. During deposition, the substrate holder rotated at a speed of 15 rpm to achieve uniform film thickness across the entire substrate.

The film thickness was measured by a Dektak 150 profilometer. The X-ray diffraction 2 theta scan for the films was acquired using a Bruker 800 234-X-ray diffractometer (9729) with a Cu tube (λ=0.154184 nm).

The TEM analysis was performed using a Thermo Fisher Spectra 300 transmission electron microscope. Cross-sectional TEM images and diffraction patterns were acquired from specimens prepared by a ZEISS Crossbeam 550 FIB-SEM system, covering the full thickness of the AlN thin films.

The film roughness was measured by a Bruker Dimension FastScan Atomic Force Microscope. Each measurement was performed over a 1 μm by 1 μm area, and three measurements were taken per sample to minimize noise and improve accuracy.

The piezoelectric coefficient d_33_ measurement was performed with a Bruker Dimension FastScan Atomic Force Microscope equipped with a platinum–iridium coated conductive tip (SCM-PIT-V2, Bruker, USA). The measurement setup and sample structure are illustrated in Figure S2. For each sample, a 300 nm thick aluminum bottom electrode was first deposited on a silicon substrate, followed by a 1 μm-thick patterned aluminum nitride (AlN) layer, deposited either with or without ion beam assistance. Silver paste was applied to establish electrical contact between the aluminum electrode and the AFM sample stage.

## Supplementary Material



## Notes

XRD measurement patterns, surface roughness characterization of AlN samples, cross-sectional SEM image of a 1 μm-thick AlN film deposited with an ion source on a silicon substrate, and schematic diagram of the piezoelectric coefficient measurement setup using piezoresponse force microscopy (PDF)
