# Supplementary material for: Broad Beam Plasma Enhanced Low-Temperature Growth of Oriented Aluminum Nitride Thin Films
Source: ACS Appl Mater Interfaces. 2025 Nov 4;17(45):62277–84. doi: 10.1021/acsami.5c13449 (PMC12616607; doi:10.1021/acsami.5c13449)
Supplement: Supplementary file 1 [file am5c13449_si_001.pdf]

Supporting Information

Broad Beam Plasma Enhanced Low-Temperature Growth of Oriented Aluminum Nitride Thin Films

Yifan Liu[1,2], Keliang Wang[3], Tyler Johnson[4], Aniwat Juhong[1,2], Junwoo Lee[4], Bo Li[1,2], Shi-You Ding[5], Zhen Qiu[1,2,6*], Qi Hua Fan[1,4*]

[1] Department of Electrical and Computer Engineering, Michigan State University, East Lansing, MI 48824, USA

[2] Institute for Quantitative Health Science and Engineering, Michigan State University, East Lansing, MI 48824, USA

[3] Fraunhofer USA Center Midwest, East Lansing, MI. 48824, USA

[4] Department of Chemical Engineering and Materials Science, Michigan State University, East Lansing, MI 48824, USA

[5] Department of Plant Biology, Michigan State University, East Lansing, MI 48824, USA

[6] Department of Biomedical Engineering, Michigan State University, East Lansing, MI 48824, USA

*Correspondence: qiuzhen@msu.edu, qfan@msu.edu

Table S1. XRD features of AlN films prepared with BIS-DCMS system

Sample number	Grow temperature /°C	Ion source DC/V	Ion source RF/mA	Thickness/ nm	(0002) Center angle/°	2θ Scan FWHM of (0002)/°	$(\mathbf{0002}):(\mathbf{10\bar{1}1})$
1	RT	NA	NA	400	36.2049	0.7298	1:4.57
2	RT	80	200		36.0326	0.4460	1:0.27
3	RT	80	400		36.1847	0.2636	1:0.31
4	RT	120	200		36.0427	0.3954	1:0.16
5	RT	120	400		36.0022	0.3751	1:0
6	200	NA	NA		NA	NA	0:1
7	200	80	200		36.1137	0.2636	1:0.395
8	200	80	400		36.1542	0.2635	1:0.372
9	200	120	200		36.1441	0.2635	1:0.077
10	200	120	400		36.0732	0.3544	1:0

Table S2. Roughness features of AlN films prepared with BIS-DCMS system

Sample number	Grow temperature/°C	Ion source DC/V	Ion source RF/mA	Average roughness (Sa)/nm	RMS roughness (Sq)/nm
1	RT	NA	NA	2.65	3.31
6	200	NA	NA	2.86	3.60
5	RT	120	400	0.95	1.25
10	200	120	400	1.23	1.54
3	RT	80	400	1.85	2.33
8	200	80	400	1.92	2.41

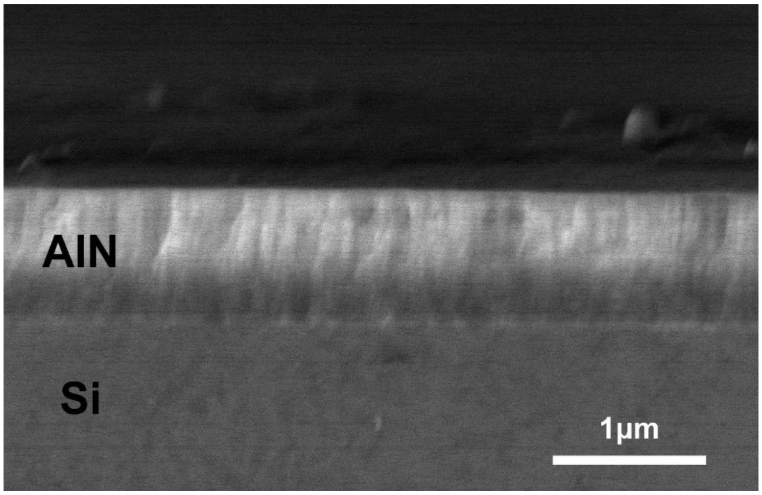

Figure. S1 Scanning electron microscopy images of 1μm AlN thin film deposited with Ion source on the silicon substrate in cross sectional view

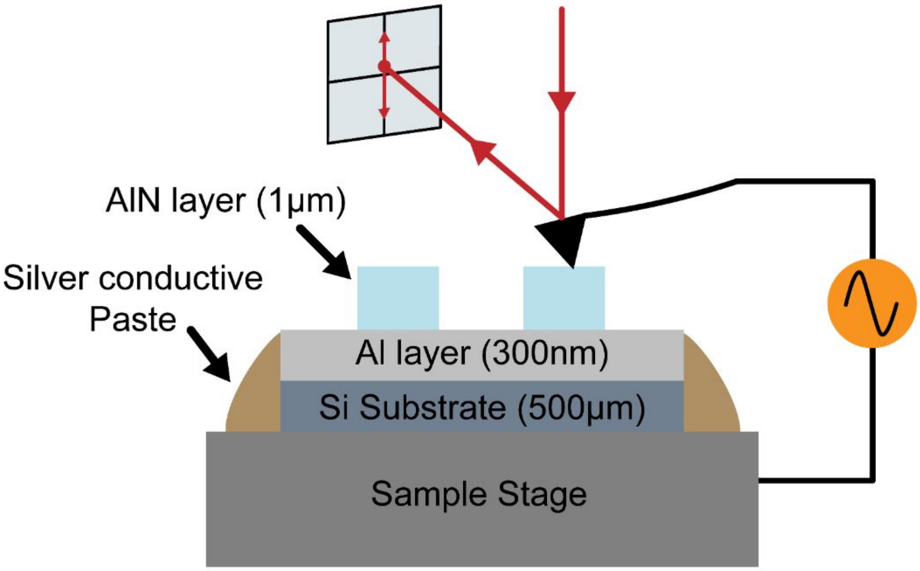

Figure. S2 Schematic of piezoelectric coefficient d_{33} measurement setup and layered sample structure
